# Safe exposure distances for transcranial magnetic stimulation based on computer simulations

**DOI:** 10.7717/peerj.5034

**Published:** 2018-06-18

**Authors:** Iam Palatnik de Sousa, Carlos R. H. Barbosa, Elisabeth Costa Monteiro

**Affiliations:** Postgraduate Program in Metrology, Pontifícia Universidade Católica do Rio de Janeiro, Rio de Janeiro, Brazil

**Keywords:** Transcranial magnetic stimulation, Safety, Spatial distribution, Exposure limits, Metrology

## Abstract

The results of a computer simulation examining the compliance of a given transcranial magnetic stimulation device to the 2010 International Commission on Non-Ionizing Radiation Protection (ICNIRP) guidelines are presented. The objective was to update the safe distance estimates with the most current safety guidelines, as well as comparing these to values reported in previous publications. The 3D data generated was compared against results available in the literature, regarding the MCB-70 coil by Medtronic. Regarding occupational exposure, safe distances of 1.46 m and 0.96 m are derived from the simulation according to the 2003 and 2010 ICNIRP guidelines, respectively. These values are then compared to safe distances previously reported in other studies.

## Introduction

Transcranial magnetic stimulation (TMS) is a therapeutic technique consisting in a rapidly changing electric current passing through a small coil placed above the scalp (Wasserman, 1998). The magnetic field generated by the coil induces currents within the brain, without significant current induction in the scalp or typical side effects of direct percutaneous electrical stimulation (Wasserman, 1998).

The pulses generated by the device can depolarize neurons and, with repeated application, modulate cortical excitability, depending on the parameters of stimulation. This has been shown to result in behavioral consequences and therapeutic potential for psychiatric conditions such as anxiety and depression (Rossi et al., 2009). The typical principal frequency of individual TMS pulses is of about 3 kHz. These individual pulses, however, are delivered with a pulse repetition frequency typically below 25 Hz (Wasserman, 1998).

This technique is currently approved by Health Surveillance Agencies of Australia, Brazil, Canada, European Union, Israel, New Zealand, Russia and USA, among others (Rossi et al., 2009; López-Ibor, López-Ibor & Pastrana, 2008; Conselho Federal de Medicina, 2012). Registration by health agencies requires that the TMS devices demonstrate compliance with various technical standards. Until now, however, no particular standard containing specific requirements for TMS devices has been published (Palatnik-de-Sousa & Monteiro, 2014). Besides a complete description of the stimulus, aspects regarding device reliability, including safety and performance checks, should be satisfactorily considered (Monteiro, 2007; Monteiro & Leon, 2015). The World Health Organization (WHO) has addressed magnetic field safety in several publications (WHO, 2006). Notably, it has encouraged the adoption of exposure limits as defined on the guidelines published by the International Commission on Non-Ionizing Radiation Protection (ICNIRP, 2003).

Considering that TMS is a technique involving the delivery of a dose of non-ionizing radiation, several concerns regarding exposure and associated relevant definitions are raised.

ICNIRP has issued guidelines for protection against magnetic and electromagnetic fields on different occasions (ICNIRP, 1998, 2003, 2010, 2014). The 1998 guideline presents requirements for a frequency range spanning from 0 Hz to 300 GHz (ICNIRP, 2003). In 2003, ICNIRP published an additional document containing information regarding procedures for testing compliance with the 1998 guideline, for non-sinusoidal and pulsed fields (ICNIRP, 2003). The 2010 and 2014 guidelines revise the recommendations for 1 Hz to 100 kHz and static fields, respectively (ICNIRP, 2010, 2014).

The magnetic and electric field limits provided in these guidelines are divided in basic restrictions (BR), and reference levels (ICNIRP, 2010). The first are based directly on established health effects. The latter are provided for practical exposure assessment given that the internal electrical field strength is difficult to assess (ICNIRP, 2010). This helps in determining whether the BR are likely to be exceeded. The ICNIRP guidelines can be used as a basis to determine which distances would be within occupational safe limits for operators, considering the exposure to the fields emitted by the TMS device (Karlström et al., 2006; Lu & Ueno, 2010).

A previous work (Karlström et al., 2006), using dimensional measurements performed with a calibrated coil and considering the magnetic field exposure limits recommended by the 2003 ICNIRP guideline (ICNIRP, 2003), found that a distance of about 0.7 m or more was considered to be safe for the TMS device evaluated, consisting of a MC-B70 coil by Medtronic Synectics with a MegPro unit (Medtronic Synectics AB, Järfälla, Sweden).

Notably, Karlström et al. (2006), provided experimental plots showing the pulse shape for the time derivative of the magnetic flux density and for the maximum value of this quantity as a function of the vertical distance to the coil center.

Later, by means of a phantom study published in 2010, Lu and Ueno identified the safe distance according to 2003 ICNIRP as being 1.1 m (Lu & Ueno, 2010). It must be noted, however, that Karlström et al. (2006) and Lu & Ueno (2010) did not evaluate the same brand and model of TMS device.

Lu & Ueno (2010) have analyzed the dependence of induced currents in a real human model, testing different coil shapes, distances between coil and human model, and rotation of the coil in space. Namely, the coils used included a figure-of-eight and a round model, of unspecified brands. The difference in coil shapes, as noted by Lu and Ueno themselves, implies a difference in magnetic flux density spatial distribution, and decay with distance.

Nadeem et al. (2003) have used the impedance method to calculate induced currents and electric field distributions on the human brain associated with TMS, using a three-dimensional human head model. The magnetic field simulated was generated with a virtual model of a figure-of-eight TMS coil MC-B70 (by Medtronic), calculated using Biot–Savart’s law. The study provides parameters regarding both coil and current shapes.

There has been no previous work comparing the experimental results of Karlström et al. (2006) and the simulation results of Nadeem et al. (2003), even though they both study the same brand and model of TMS coil. Reconciling the independently achieved empirical and theoretical results would allow for a better understanding of the distribution of the magnetic flux densities, and of the safe distances regarding this device.

However, the published simulation data by Nadeem et al. (2003) does not include the variation of the magnetic flux density as a function of distance on the same axes as the results of Karlström et al. (2006).

As such, this manuscript aims at revisiting the safe distance values given in literature in light of the more recently published ICNIRP guidelines (ICNIRP, 2010), and discussing some key aspects of previous studies on the theme while also providing new data for consideration. By generating a new simulation of the magnetic flux densities that includes how the fields decay on 3D directions, all of the gaps left in the comparison of the previous empirical and theoretical results from literature could be addressed.

For this, the research began by simulating the fields generated by the MC-B70 coil (Karlström et al., 2006; Nadeem et al., 2003). Using the parameters given in literature (Lu & Ueno, 2010), as a basis for TMS coil and pulse shapes, the study intends to compare the simulated data by reaching, through computational means, the same conclusions achieved by the measurements provided by Karlström et al. (2006).

The final objective is to use the new simulation results compared to previous empirical data and the recently published 2010 ICNIRP guidelines (ICNIRP, 2010) to derive more robust estimates for the safe distance.

## Materials and Methods

The simulation of the magnetic field generated by the MCB-70 coil was based on several MATLAB (MATLAB®; The MathWorks, Natick, MA, USA) routines that were developed in this study to define the parameters for the calculation of Biot–Savarts’s law, the coil and current shapes, among others. The figure-of-eight coil consists basically in a pair of spiral coils (Coil_A_ and Coil_B_) at an angle, with folds towards the center.

**Figure 1:**
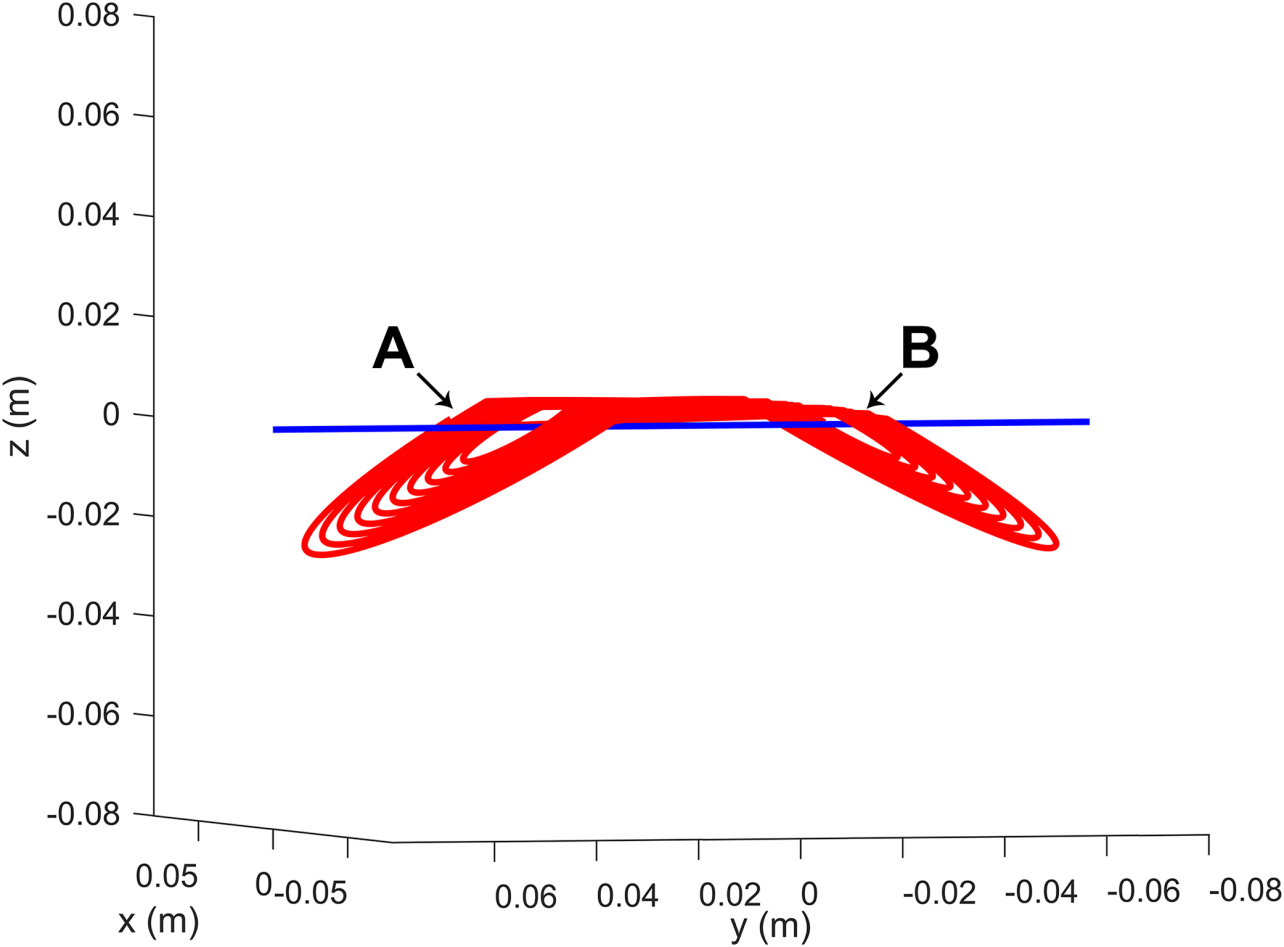
Virtual model of the MCB-70 coil used in the simulation. The coil system is comprised of two coils, Coil_A_ and Coil_B_, which are illustrated and indicated above in A and B, respectively. The blue line represents the axis along which the fields seen in Fig. 2 were simulated.

Figure 1 shows the final shape of the coil used for the simulations, as well as the axes defined. Coil_A_ is located to the left of the figure, slightly above Coil_B_, which can be seen to the right.

Using the parameters provided in Nadeem et al. (2003), Coil_A_ and Coil_B_ were generated by first using the parametric Eqs. (1–3), with the angular variable θ to define a spiral of Archimedes with center at (0,0,0), with inner radius (*r*_inner_) of 1 cm and the variable *p* being used to ensure an outer radius (*r*_outer_) of 5 cm on the *x–y* plane. The number of turns in the coil winding is defined by the variable *n* such that the total number of turns is 10.

$$x = ({r_{\rm inner}} + p{\rm{\theta }})\cos \left({-{\rm{\theta }}} \right)$$ (1)

$$y = ({r_{\rm inner}} + p{\rm{\theta }})\sin \left({-{\rm{\theta }}} \right)$$ (2)

$$z = 0$$ (3)

$$n = 10$$ (4)

$$p = {{{r_{\rm{outer}}}-{r_{\rm{inner}}}} \over {2\pi n}}$$ (5)

$$\theta = \left[ { - 2\pi n;0} \right]\left( {\rm{coil}}_{\rm {A}} \right){\rm{}};\left[ {2\pi n;0} \right]\left( {{\rm{coil_B}}} \right)$$ (6)

This spiral was then rotated around the *x*, *y* and *z* axes. Namely, the only rotations used in this case were 212° (32° + 180°) and −32° around the *x*-axis with each value guaranteeing that both coils would end up at a 32° inclination. However the variable θ has opposite ranges for both coils, causing them to be wound up in opposing directions. This ensures the fields from Coil_A_ and Coil_B_ have *z* components pointing in opposite directions, as is the general case for TMS coils.

The center of the rotated spiral coils then underwent a translation to their final positions. A cut-off value was set for the *z* variable, defining the height above which the coils fold. This fold is accomplished by rotating back the portion of the spirals above the cut-off height. The result can be seen in Fig. 1.

The full list of parameters to generate the coils comprise not only the already cited inner radius, outer radius, coil center coordinates and rotation angles around the three axes, but also the vertical (*z*-axis) and horizontal (*y*-axis) distances between the coil centers.

Namely, the area above the convex side of the coil, towards the positive values of the *z*-axis, will be referred to as “operator side” of the coil, whereas the area below the concave side, towards the negative values of *z*, will be called the “patient side” of the coil.

A numerical integration is then performed along the path defined by the coil shape, calculating the three components (*B_x_*, *B_y_* and *B_z_*) of the magnetic flux densities by the Biot–Savart’s law. The distance between Coil_A_ and Coil_B_ was varied until the spatial distribution of the magnetic flux densities had a similar shape, with peaks of close values located in similar positions to those presented by Nadeem et al. (2003).

Another procedure was used to define the shape of the current pulse. The currents basically consist of trains of single sinusoidal pulses separated by given intervals.

The full list of parameters that define the current shape include: the period (duration) of the sinusoidal pulses, which define their frequency, the amplitude of these pulses, the total duration of the trains, the pulse repetition frequency, the inter-train and inter-pulse intervals and the number of pulses in a train. Notably some of these quantities are fully defined by the values of the others, but they were still defined as separate variables to allow for a clearer understanding.

The choice for sinusoidal pulse shapes was made based on the pulse shapes obtained by Karlström et al. (2006) in their measurements and by Nadeem et al. (2003) in their simulations.

In the present simulation, the current consisted of pulses of 7.66 kA amplitude, 286 μs duration (∼3.5 kHz equivalent frequency), 5 Hz pulse repetition frequency, 1 s train duration and 5 s inter-train interval. The inter-pulse interval is determined from the variables above.

An adapted version of the Biot–Savart calculating routine then performed the calculations as a function of time. This was done with the same coil shape parameters, but considering the instantaneous value of the current in each iteration of the time loop in order to calculate the magnetic flux density at a given point in space. The result was the temporal distribution of the magnetic flux density for a point in space specified by the user.

Calculating the Biot–Savart’s law results at each temporal step, considering the instantaneous value of the current, is an approximation, given that the natural time varying generalization of Biot–Savart’s laws are the Jefimenko equations (Griffiths & Heald, 1991). However, these would be harder to simulate as the temporal integrals involved are notably more complex, and it was possible that, at the frequency range used in this case, the Biot–Savart approximation used would be sufficient. The latter results would come to show this to be true. Finally, the time derivative of the magnetic flux density was computed through numerical differentiation in order to obtain the d*B*/d*t* signal. Notably, the magnetic flux density given by the simulation follows the same sinusoidal character of the current pulses, with the same frequency of about 3.5 kHz, and the same happens for d*B*/d*t* (with a 90° phase caused by the differentiation). Thus one may estimate the behavior of the amplitude of d*B*/d*t* as a function of distance by multiplying the amplitude of *B* for those distances by the value of the angular frequency, since the amplitude of the derivative of a sinusoidal function is simply the amplitude of the original function multiplied by its angular frequency.

Both the d*B*/d*t* pulse shape for this quantity and its amplitude were compared to the experimental results reported in 2006 by Karlström et al. (2006). Notably, the data from the log scale d*B*/d*t* vs distance plot was extracted from the graph published in Karlström et al. (2006) using a data digitizing software (Gsys) to allow for closer, albeit approximated, comparison with the simulation.

Both sets of data were compared. From these values it was possible to extrapolate for the occupational exposure safe distance values following the procedure described by Karlström et al. (2006), which mainly consists in considering the 3.5 kHz component of the pulse as the only contribution and using this as a basis to perform the calculations according to the ICNIRP 2003 guidelines (ICNIRP, 2003). As a comparison, the safe distance was also determined by directly verifying the distance below which the simulation data reached the acceptable value for d*B*/d*t*, eliminating the need for the extrapolation described in Karlström et al. (2006).

In a similar manner, the calculations suggested by the 2010 guideline were performed using the simulation data and the result was compared to the safe distance of about 0.7 m proposed by Karlström et al. (2006). The raw data generated by the simulation is provided as a supplemental file alongside this manuscript.

## Results

**Figure 2:**
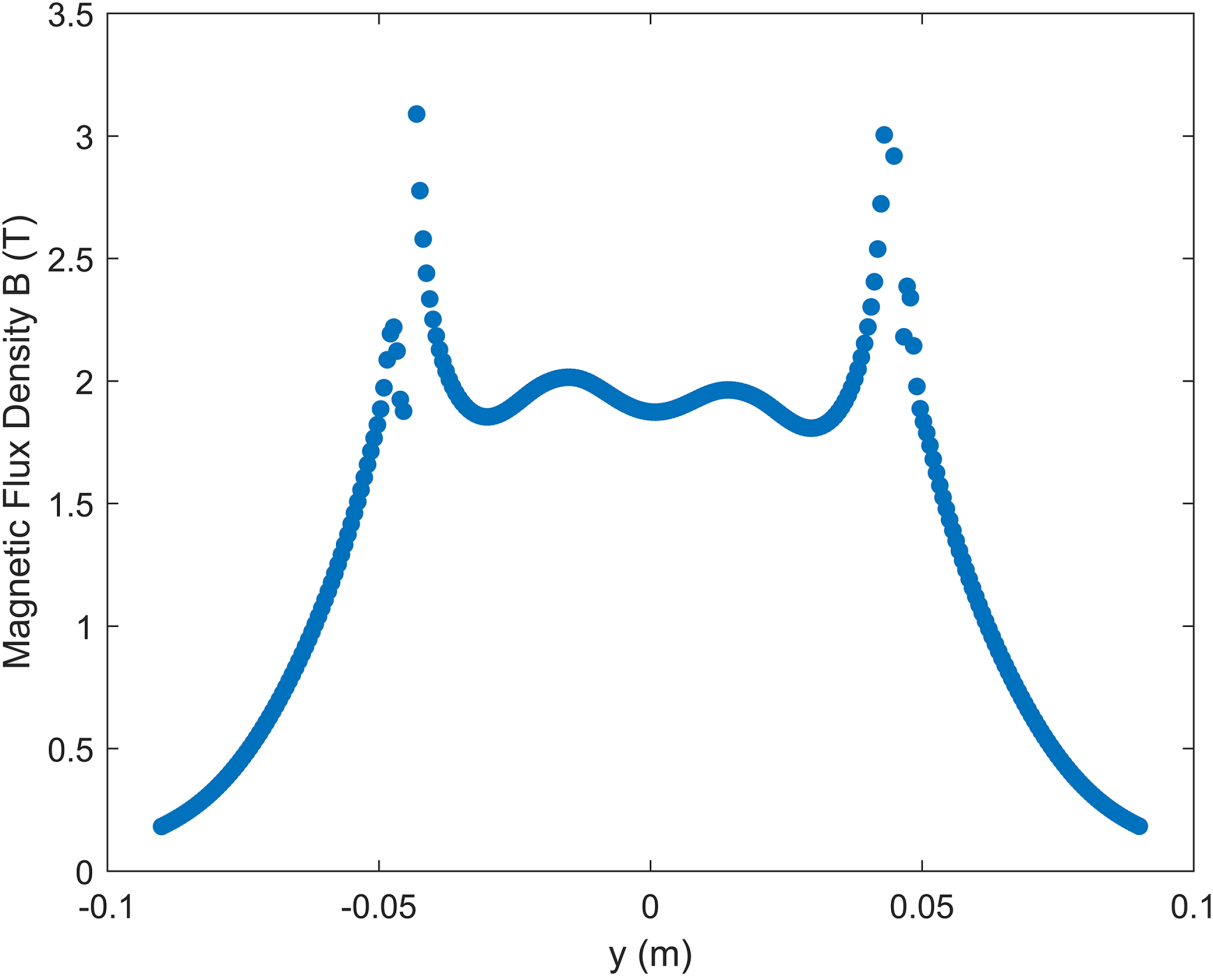
Dependence of the magnitude of the magnetic flux density |*B*| with the *y* coordinate, at *x* = 0 and a distance of 8.3 mm below the surface of the coil.

Figure 2 shows the dependence of the magnitude of the magnetic flux density (|*B*|) with the *y* coordinate. These values were obtained after varying the coil shape parameters and the vertical distance of the coil to the *x–y* plane until a satisfactory match to the simulation data in literature (Nadeem et al., 2003) was found. The general curve shape and the position and value of the peaks found at a distance of 8.3 mm below the coil center are close to the ones found by Nadeem et al. (2003) for distances between 5 and 10 mm from the coil surface. Thus, this vertical distance of 8.3 mm between coil and *y*-axis was selected for the simulation. This difference of about 3 mm and other slight differences can be associated with further details in how the coils were modeled in each simulation, and on how the 0 mm height was defined in Nadeem et al. (2003). Notably, the expression “at the coil surface” present in Karlström et al. (2006) and Nadeem et al. (2003) can generate some ambiguity.

**Figure 3:**
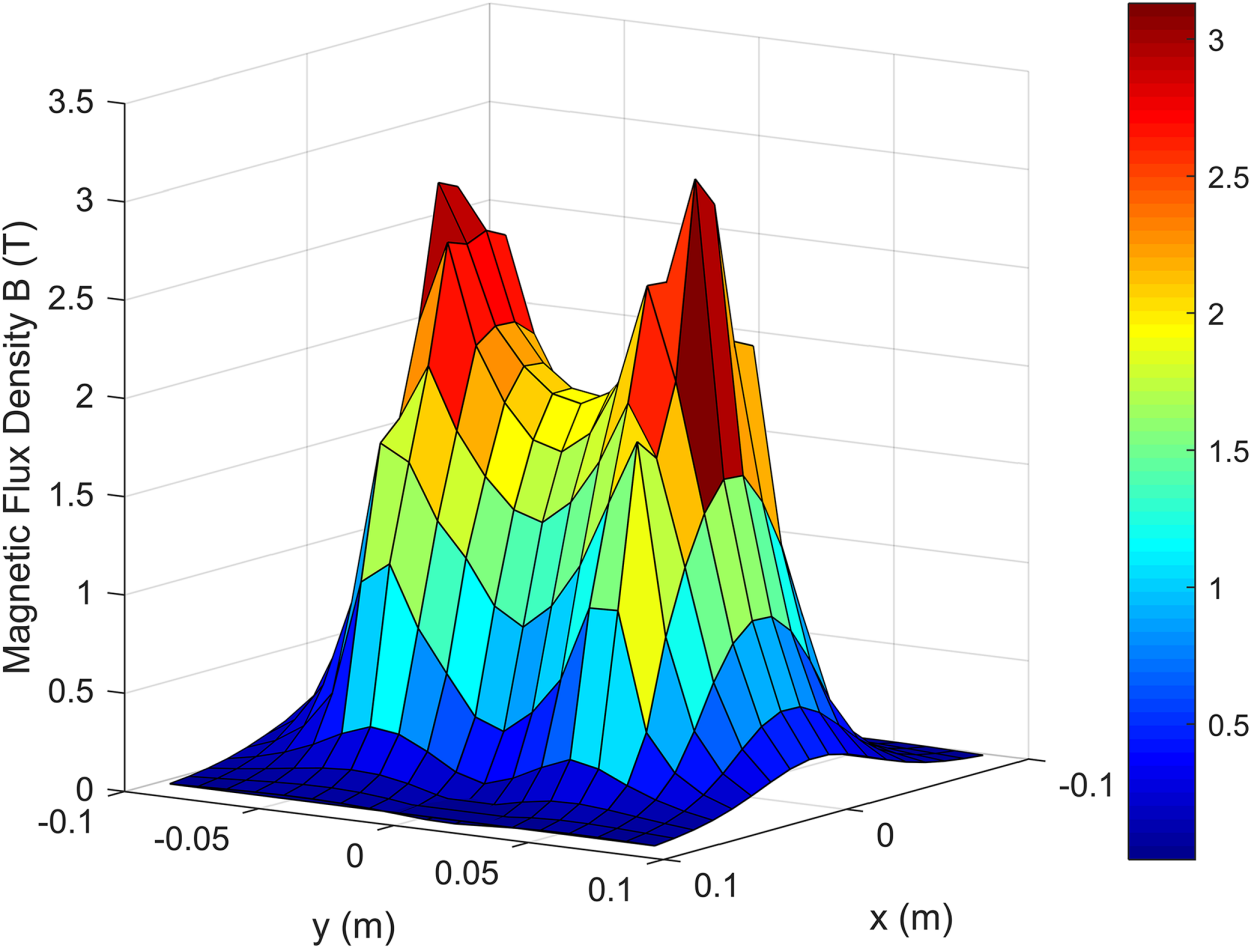
Spatial distribution of *B* on the *x*–*y* plane, 8.3 mm below the surface of the coil.

By generalizing the calculations to the whole *x*–*y* plane, the spatial distribution shown in Fig. 3 could be obtained, showing how the magnetic flux density behaves around the coil area.

**Figure 4:**
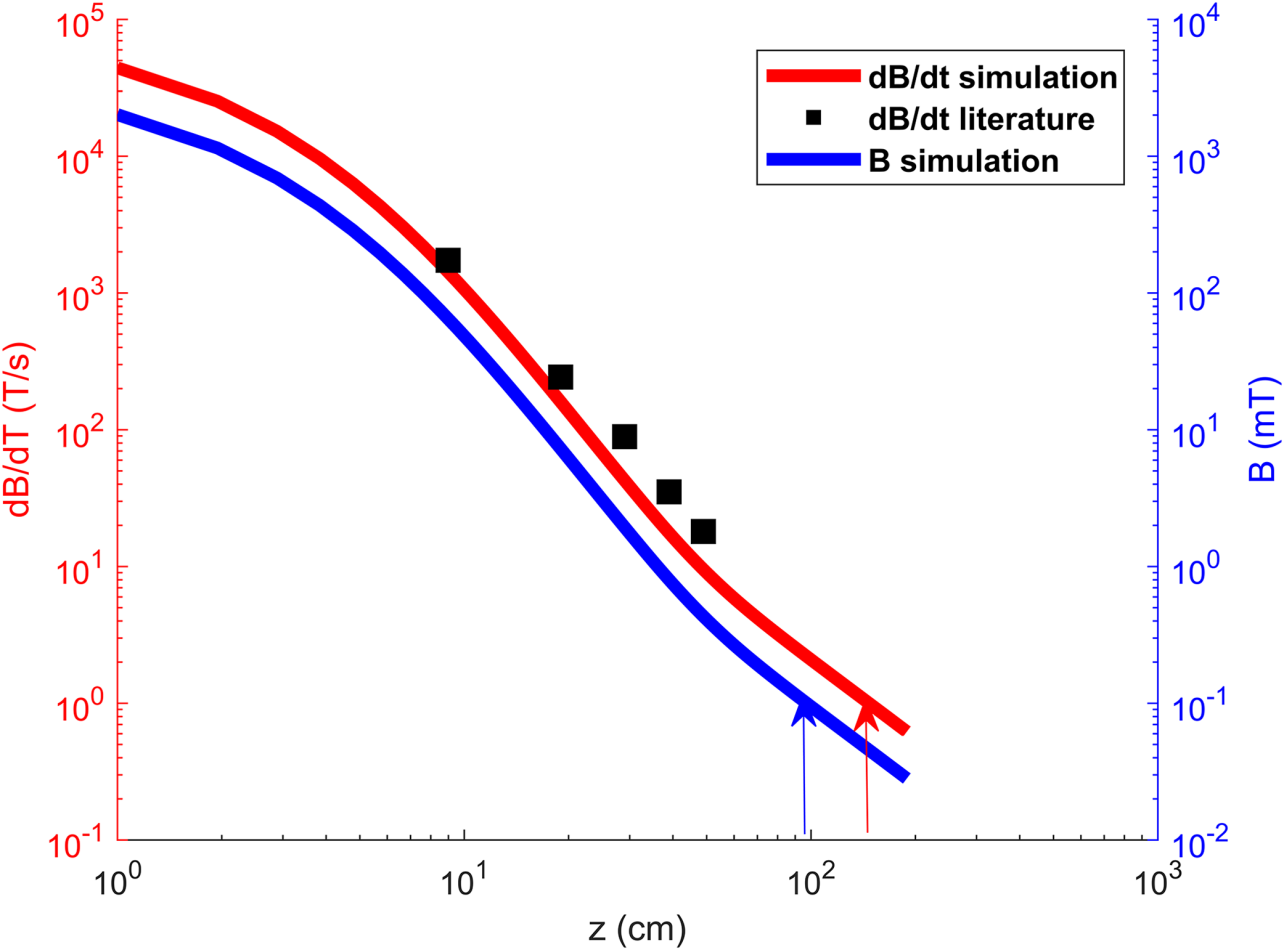
*B* and d*B*/d*t* as a function of the distance on the *z*-axis (operator side of the coil). Red and blue curves and axis refer to d*B*/d*t* and *B* values, respectively. Red and blue arrows show the safe distance values obtained by direct comparison of simulation data with the limits of 1 T/s and 0.0001 T provided for d*B*/d*t* and *B* by the 2003 and 2010 ICNIRP guidelines, respectively. The black squares indicate the measured d*B*/d*t* values available in literature Karlström et al. (2006) and show how the simulation closely approximates these experimental results.

The comparison between the present simulation data and the experimental results of Karlström et al. (2006) is shown in Fig. 4, where the upper curve (in red) corresponds to the left vertical axis with d*B*/d*t* values and the lower curve, in blue, corresponds to the right vertical axis with the magnetic flux density values. Both curves were obtained from simulation data. The black squares indicate data extracted from the results of Karlström et al. (2006).

The safe distances, indicated in Fig. 4 by arrows, were calculated using methodologies of the ICNIRP (2003), provided for d*B*/d*t*, and 2010 (ICNIRP, 2010), for *B* values, considering that the magnetic field has a ∼3.5 kHz (pulse duration of 286 μs) fundamental frequency that allows disregarding the other components, similarly to the treatment performed by Karlström et al. (2006).

The safe exposure is, then, associated with a magnetic flux density of 0.1 mT, according to the ICNIRP 2010 guideline (ICNIRP, 2010). This result can be achieved by maintaining a distance of about 96 cm to the operator’s side of the coil’s surface, as seen by direct inspection of the simulation data shown in Fig. 4.

By computing the base 10 logarithms of d*B*/d*t* and of the distances provided by Karlström et al. (2006), a linear fit provided an intercept of 6.05 with a slope of −2.81. The adjustment *R*^2^ was 0.9985.

Using the d*B*/d*t* limit value of 1 T/s considered by Karlström et al. (2006) and recommended by the 2003 ICNIRP guideline (ICNIRP, 2003), one may obtain the safe distance (*d*_safe_) by extrapolating the behavior of the data until reaching the distance where log(d*B*/d*t*) has a value of zero (since d*B*/d*t* has a value of 1 T/s), as can be seen in Fig. 4. From the fitted line equation, one may deduce that this distance is then given by

$$\log \left({{{dB} \over {dt}}} \right) = Slope{\rm\,\,{*}}\,\log \left(d \right) + Intercept$$ (7)

$$0 = Slope{\rm\,\,{*\,\,log}}\left({{d_{\rm safe}}} \right) + Intercept$$ (8)

$${d_{\rm safe}} = {10^{{{\rm Intercept} \over {\rm -Slope}}}}$$ (9)

which gives a safe distance of approximately 1.43 m for these fitting parameters.

One may also use the simulation data to obtain through direct inspection that distances about 1.46 m are safe, as seen in Fig. 4, without using Karlström et al.’s extrapolation.

## Discussion

The dependence of d*B*/d*t* with the distance to the coil surface (on the operator side) obtained with the present simulation (Fig. 4), closely resembles the measurement results shown in Fig. 1 of reference (Karlström et al., 2006). However, the simulated data seems to be offset by about 0.8 to 3 cm on the *z*-axis when compared to the measurements.

Most likely this stems from the definition of what “at the coil surface” means. This is an expression used both in Karlström et al. (2006) and Nadeem et al. (2003), remaining unclear as to how this surface is defined and if the concept is used similarly for measurements on both sides (operator and patient). This might also explain why the simulation shown in Fig. 2, at 8.3 mm below the coil center, shows values between those found by Nadeem et al. (2003) for distances of 5 and 10 mm below the coil surface. The exact definition of the 0 cm height is ambiguous in literature.

The simulated fields have to take into account that measurements were most likely performed on the surface of the plastic chassis that surrounds the wire winding, and not exactly on the surface of the wires. Offsetting all the simulated data points by about 3 cm could be justified, then, as a correction to try to consider the influence of the external plastic chassis. Another influence that might factor in would be a systematic error on the definition of the surface level (which would represent a distance of 0 cm to the coil surface), on the reported measurements. Since the points seem to be offset by a constant value, this could also be a possible explanation for the differences. Thus, it is possible to assume that the simulated data fits the measurements closely.

The 2003 and 2010 ICNIRP guidelines mention that a spectral analysis is indicated for signals with more than one frequency component. However, the TMS magnetic flux density, over time, has a pulse behavior similar to the current. Since the variation of this quantity happens only during the pulses (being zero elsewhere), and these are cycles of a regular 3.5 kHz sinusoidal wave, this may justify treating the exposure only considering a 3.5 kHz component, as done by Karlström et al. (2006) and regarding the ICNIRP calculations (ICNIRP, 2003). Furthermore, the pulses are usually single, whereas the 2010 ICNIRP guidelines define them as having at least five cycles (ICNIRP, 2010), which further justifies leaving the spectral analysis aside, even if this would constitute a first approximation. Future studies might look further into ways of spectrally decomposing this signal to test possible effects of other harmonics, and of the different precautions that might be taken for different pulse repetition frequencies, if necessary. This, however, would require defining how many pulses, or trains of pulses, should be considered for the evaluation.

Using the data extracted from the d*B*/d*t* vs distance plot in Karlström et al. (2006) (shown here in Fig. 4), a safe distance of 1.43 m was obtained through an extrapolation as described by Karlström et al. (2006). However, by directly inspecting the simulation data shown in Fig. 4, d*B*/d*t* reaches values of about 1 T/s at distances around 1.46 m (*d*_safe_ according to 2003 ICNIRP), and *B* reaches values of 0.1 mT at about 0.96 m (*d*_safe_ according to 2010 ICNIRP). These results show that the most recent guidelines provide a less conservative safe distance for occupational exposure in this case.

Any documents that suggest the safe distance of 0.70 m (most likely based on the studies of Karlström et al. (2006)) should be updated with these new, more conservative, estimates. One example of such a document would be the Russian existing certificate of compliance to the СанПиН 2.2.4.1191-03 standard for a given TMS device of another brand, different than MC-B70, but also based on a figure-of-eight coil. On that certificate the distance of 0.7 m is suggested for the device operators.

Despite the difference in models, the presently indicated estimates are the best starting point for occupational safe distance considerations, as they are more conservative.

## Conclusions

A simulation of the typical magnetic flux densities generated by the MCB-70 coil was performed, and the resulting spatial distribution was used for estimation of safe distances regarding exposure, considering the ICNIRP guidelines (ICNIRP, 2003, 2010). The results of the simulation were compared to the linear experimental results available in the literature (Karlström et al., 2006).

Safe distances of 1.43, 1.46 and 0.96 m have been found by three different methodologies. For the first *d*_safe_ (1.43 m), an extrapolation similar to the one performed by Karlström et al. (2006), considering the limit of 1 T/s for values of d*B*/d*t* according to 2003 ICNIRP recommendations (ICNIRP, 2003), was used. For the second *d*_safe_ (1.46 m), a direct inspection of d*B*/d*t* as a function of distance was evaluated (Fig. 4), also considering the limit of 1 T/s given by ICNIRP (2003). The third *d*_safe_ (0.96 m) was obtained by a direct inspection of the values of *B* as a function of distance (Fig. 4), considering the limit of 0.1 mT given by 2010 ICNIRP recommendations (ICNIRP, 2010).

Since the present simulation was compared with empirical data available in literature (Karlström et al., 2006) and allows predicting the values of *B* and d*B*/d*t* for several distances, the estimate given by the linear extrapolation might be overly conservative. Although the *d*_safe_ of 0.96 m found is close to the value of 1.10 m obtained for both a figure-of-eight and a round TMS coil of non-specified brand by Lu and Ueno, based on 2003 ICNIRP, the value of 1.46 m is safer and could be the more conservative alternative adopted.

Thus, documents and studies dealing with operator safety for TMS devices can enhance safety by suggesting the usage of 1.46 m value for *d*_safe_.

Future studies can validate and enhance this result through experimental measurements of several models and brands of TMS devices, also considering the impact of associated measurement uncertainties (Palatnik-de-Sousa & Monteiro, 2015). This would allow for a comparison on how *d*_safe_ might vary in those cases and there might be a more appropriate value when considering several different brands. However, the value of 1.46 m can be seen as an initial option that is more cautious than previous estimates given in literature.

## Supplemental Information

10.7717/peerj.5034/supp-1 Supplemental Information 1 Data used for plot generation and safe distance estimation.

## Additional Information and Declarations

### Data Availability

The following information was supplied regarding data availability: The data for the plots are provided as an OPJ file that can be viewed using the freely available OriginViewer software: https://www.originlab.com/viewer/.
